# Acute Obstructive Choledocholithiasis: A Case of Elusive Gallstones on Imaging

**DOI:** 10.7759/cureus.8489

**Published:** 2020-06-07

**Authors:** Karolina N Dziadkowiec, Larnelle N Simms, Sweet Gerlie A Smith, Aviv Katz, Kayode Olowe

**Affiliations:** 1 Internal Medicine, University of Miami, John F. Kennedy Regional Campus, West Palm Beach, USA; 2 Internal Medicine, University of Miami, John F. Kennedy Regional Campus, Atlantis, USA; 3 Gastroenterology, University of Miami, John F. Kennedy Regional Campus, Atlantis, USA

**Keywords:** choledocholithiasis, endoscopy, biliary, gall stones

## Abstract

Acute choledocholithiasis results when stones form in the gallbladder and then pass into the common bile duct, where they may become lodged and cause obstruction. To our knowledge, very few cases are reported in which multiple imaging techniques had failed to detect the presence of gallstones, as per current literature review. We report a case of a 73-year-old woman with nausea, vomiting, and jaundice who was found to have choledocholithiasis with negative imaging on abdominal ultrasound (US), CT, and magnetic resonance cholangiopancreatography (MRCP).

## Introduction

The prevalence of cholelithiasis is approximately 20.5 million (6.3 million men and 14.2 million women) in the United States [1]. Between 5% and 30% of patients with cholelithiasis develop concomitant choledocholithiasis [2]. The diagnosis of choledocholithiasis is made based on the clinical signs and symptoms, results of liver function tests, and imaging findings. Definitive diagnosis commonly requires the use of advanced imaging, consisting of magnetic resonance cholangiopancreatography (MRCP), endoscopic retrograde cholangiopancreatography (ERCP), ultrasound (US), and CT among other imaging modalities. The best modality for the diagnosis of choledocholithiasis is controversial, as each modality has benefits and shortcomings.

## Case presentation

A 73-year-old woman, with a history of hypertension and hypothyroidism, presented to the ED with a two-day history of nausea and vomiting, following meals. Associated symptoms included bloating, mild epigastric discomfort, anorexia, and choluria. The patient denied any diarrhea, constipation, sick contacts, recent travel, alcohol, tobacco, or illicit drug use.

Physical examination was notable for tachycardia and icteric sclerae. Abdominal exam was unrevealing including negative Murphy’s sign and absent organomegaly. On admission, laboratory testing showed elevated white blood cells (WBC) 20TH/uL, total bilirubin 4.1 mg/dL, alkaline phosphatase (ALP) 147 u/L, aspartate transferase (AST) 233 u/L, and alanine transferase (ALT) 177 u/L. Lipase was within normal range. Hepatitis serologies were negative. Urinalysis was positive for urobilinogen.

**Figure 1:**
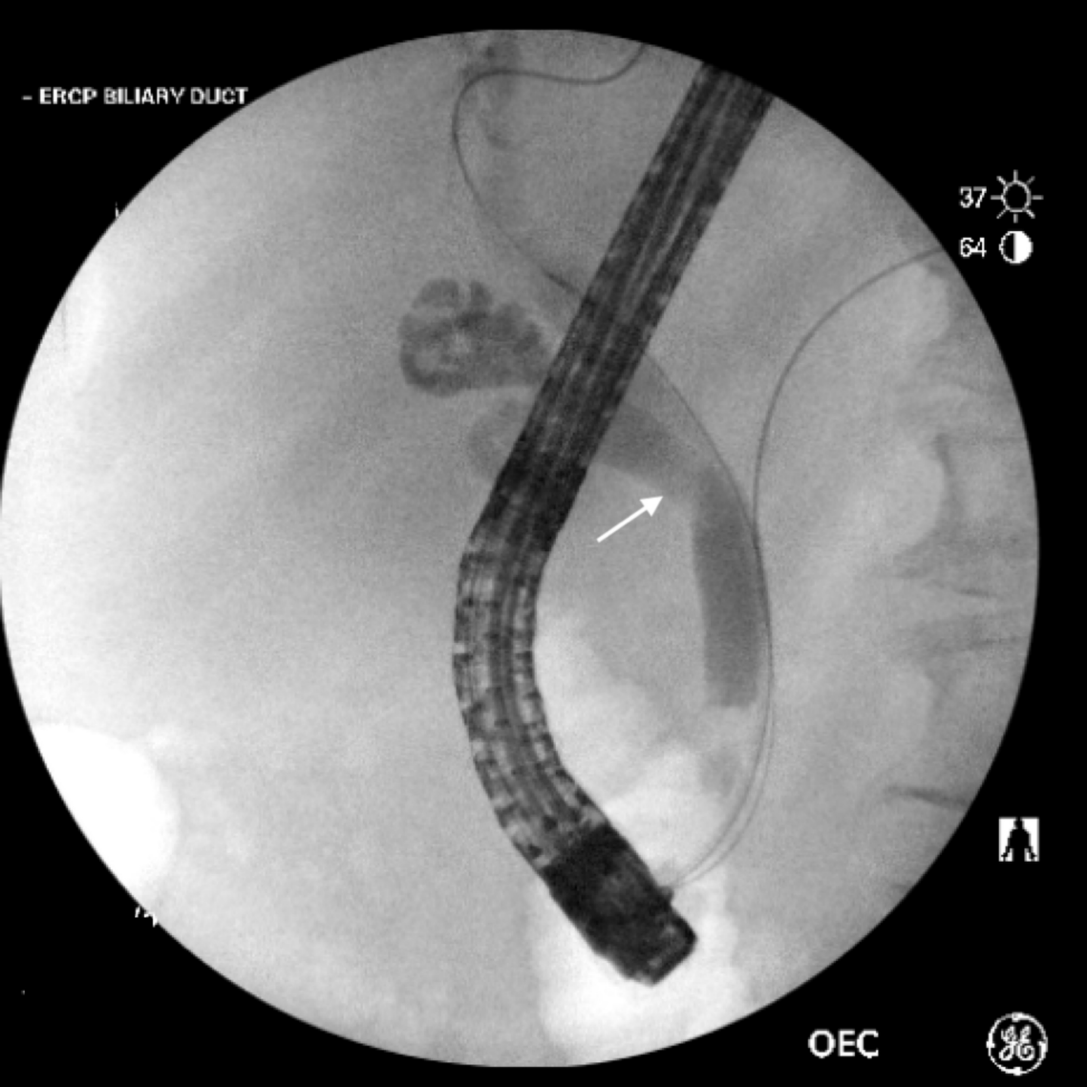
ERCP revealing a filling defect (white arrow) consistent with a stone seen on cholangiogram. ERCP, endoscopic retrograde cholangiopancreatography

Abdominal ultrasound was obtained and was negative for gallbladder wall thickening, gallstones, or intrahepatic biliary duct dilatation but the common bile duct was reported as borderline dilated at 0.63 cm. Based on these results, the patient underwent abdominal CT which revealed minimal fat stranding adjacent to the gallbladder with no gallstones visualized. Due to the concerning nature of symptoms, MRCP was performed, revealing a thick-walled hydropic gallbladder with surrounding inflammation, without gallstones. As a result of the concerning clinical findings and high level of suspicion, an ERCP was obtained and revealed a filling defect and mild dilation of the entire common bile duct secondary to a gallstone obstruction (Figure 1). Biliary sphincterotomy was performed followed by balloon extraction of two stones. General surgery was consulted during the same hospitalization and the patient underwent laparoscopic cholecystectomy. The patient had no postoperative complications and was ultimately discharged home. The patient has had no further complications since hospitalization and is reported to be in good health at the time of writing this case report. 

## Discussion

The clinical and laboratory diagnosis of acute choledocholithiasis is difficult. Elderly patients are known to possibly lack the findings of Charcot’s Triad, which classically comprises abdominal pain, jaundice, and fever. Some studies suggest only 15.6% of younger and 18.8% of elderly patients as found to have the classic components of Charcot’s Triad [3]. An extensive search of the available literature has indicated very few, if any cases where multiple imaging techniques had failed to detect the presence of gallstones. 

Our patient was found to have acute choledocholithiasis in spite of false-negative imaging including abdominal ultrasound, abdominal CT, and MRCP. Based on these findings and a high clinical suspicion an ERCP was performed resulting in the removal of two stones with nonpurulent golden-brown bile noted after biliary sphincterotomy and balloon extraction.

The MRCP provides extensive detail of the biliary tract and has a sensitivity of 81%-100% and a specificity of 92%-100% in detecting choledocholithiasis [4]. Ultrasound is believed to have a sensitivity that varies between 55% and 91% in detecting choledocholithiasis -- likely a result of being highly operator-dependent [5]. CT abdomen is noted to be have a sensitivity as high as 88% for detecting choledocholithiasis [6]. When these testing modalities are combined, their additive sensitivity and specificity is even greater. ERCP combines preoperative diagnosis with management and therapeutic intervention of choledocholithiasis with stone removal. A drawback of ERCP is the risk associated with an invasive procedure including feared and potentially lethal complications such as bleeding, post-ERCP pancreatitis, cholangitis, and bowel perforation [7]. As a result, confirming the presence of choledocholithiasis with less invasive imaging prior to performing ERCP is most desirable. 

This case demonstrates the need for increased awareness when analyzing and interpreting negative findings on the appropriate initial radiological investigations. 

## Conclusions

The diagnosis of choledocholithiasis is confirmed with advanced imaging techniques, including MRCP and ERCP. Given the various diagnostic and therapeutic options available for managing choledocholithiasis, clinical interpretation and assessment are paramount. Difficult stone burden and difficult biliary anatomy may require advanced surgical, endoscopic, and percutaneous techniques to extract biliary stones. This case demonstrates the importance of maintaining a high-degree of clinical suspicion and utilizing noninvasive techniques when attempting to confirm the presence of choledocholithiasis.
